# Recurrent Metatarsal Fractures in Postmenopausal Woman With Low Serum Alkaline Phosphatase: A Rare Diagnosis Not to Miss

**DOI:** 10.1177/2324709617718851

**Published:** 2017-07-06

**Authors:** Umair Iqbal, Hafsa Anwar, Ahmad Chaudhary, Madiha Alvi, Amy Freeth

**Affiliations:** 1 Bassett Medical Center, Cooperstown, NY, USA; 2 Dow University of Health and Sciences, Karachi, Pakistan

**Keywords:** hypophosphatasia, recurrent metatarsal fractures, asfotase alfa, TNSALP, alkaline phosphatase, osteomalacia

## Abstract

Hypophosphatasia (HPP) is a rare inborn error of metabolism due to a loss-of-function mutation in the gene for tissue nonspecific isoenzyme of alkaline phosphatase (ALP) that results in low levels of ALP. The clinical presentation of HPP is variable and in adults can easily be misdiagnosed as other forms of osteomalacia. We present a case of a 53-year-old Caucasian female who was evaluated for recurrent metatarsal fractures. She reported her first metatarsal fracture at age 21, and since then had at least 8 more metatarsal fractures over her lifetime, most without injury other than weight bearing. She reported history of gait disturbance as a child and dental issues (spacing and loosening). Laboratory tests showed normal serum calcium, phosphorus, and parathyroid hormone, but low serum ALP <20 IU/L and elevated N-telopeptide. Foot X-ray showed several healed and nonhealed metatarsal fractures, and bone densitometry revealed osteopenia. She was treated with calcium and vitamin D. A year later she had a new metatarsal fracture and a nontraumatic pelvic fracture. Teriparatide therapy was attempted but not tolerated. Due to suspicion of HPP vitamin B_6_ levels were checked and found to be elevated at 263 µg/L. Given her clinical presentation and low ALP levels with elevated vitamin B_6_, the diagnosis of HPP was made. Clinicians should be attentive to a history of recurrent low trauma fractures, premature loss of deciduous teeth, and persistently low serum ALP to suspect this diagnosis. Early case detection with the availability of recent Food and Drug Administration–approved asfotase alfa may avoid years of undiagnosed morbidity.

## Introduction

Hypophosphatasia (HPP) is a rare inborn error of metabolism due to a loss-of-function mutation in the gene for tissue nonspecific isoenzyme of alkaline phosphatase (TNSALP) that results in low levels of ALP.^1,2^ Over 300 mutations have been reported in the TNSALP gene, which is mostly expressed in the liver, skeleton, and developing teeth. Due to these mutations, substrate of TNSALP inorganic pyrophosphate accumulates extracellularly, which leads to inhibition of bone mineralization.^1,2^ TNSALP is expressed ubiquitously, and its physiological role is evident in bone mineralization, a deficiency that can manifest in many ways, including rickets or osteomalacia. Clinical presentation is wide-ranging from nearly unmineralized skeleton resulting in death in utero to just dental manifestations of premature loss of deciduous teeth without any skeletal involvement.^1-5^ Some clinicians regarded HPP as having the broadest expressivity of all skeletal disease.^2^ We present a case of a middle-aged woman evaluated for recurrent metatarsal fracture diagnosed with HPP.

## Case

A 53-year-old Caucasian female was evaluated for recurrent metatarsal fractures. She reported her first metatarsal fracture at age 21, and since then had at least 8 more metatarsal fractures over her lifetime, most without injury other than weight bearing. She reported history of gait disturbance as a child and was evaluated by an orthopedic surgeon for leg braces. She also reported dental issues as a child, which includes spacing and loosening of teeth. She denies any use of herbal medications. There was no history of corticosteroids use. Laboratory tests showed serum calcium of 9.8 mg/dL (8.4-10.2 mg/dL), parathyroid hormone of 34 pg/mL (14-72 pg/mL), low serum ALP <20 IU/L (38-135 IU/L), and high urinary levels of bone turnover marker N-telopeptide, which was 92 nmol/mmol (19-63 nmol/mmol) done on initial evaluation at least 6 months apart from last fracture. Foot X-ray showed several healed and nonhealed metatarsal fractures. Dual-energy X-ray absorptiometry scan was done that showed *T* score of −1.31, −2.07, and −1.88 at spine, femoral neck, and total hip, respectively. She was initially treated with calcium and vitamin D. A year later she had a new metatarsal fracture and a nontraumatic pelvic fracture. She had adverse drug reactions to denosumab, teriparatide, and bisphosphonates. She developed rash and trouble breathing with bisphosphonates. She had similar adverse effects with denosumab. She developed sinus infection after teriparatide infusion. Due to low serum ALP and history of gait disturbances as a child, a diagnosis of HPP was considered. Laboratory tests revealed elevated vitamin B_6_ (pyridoxal 5′-phosphate) levels of 263 µg/L (5-50 µg/mL) and inorganic phosphorous of 5.8 mg/dL (2.5-4.9 mg/dL). Patient denies taking any vitamin B_6_ supplementation. Given her clinical presentation along with low ALP levels and elevated vitamin B_6_, the diagnosis of HPP was made.

## Discussion

HPP was first described by Canadian physician John C. Rathbun in an infant who presented with rickets and seizures at 2 months of age with low ALP, which was discovered later in autopsy.^6^ The highest prevalence of HPP is reported among Mennonites in Manitoba, Canada, with 1 in 25 individuals as carriers of the mutation and 1 in 2500 neonates having life-threatening HPP.^7^ More than 300 mutations have been described in TNSALP gene that can be transmitted autosomal recessive (severe form) or dominant resulting in HPP (mild form).^3^

In the review of literature, at least 7 types of HPP have been described. These include perinatal, infantile, childhood (juvenile), and adult depending on the age of presentation.^3^ In general, the earlier the presentation and lower the ALP levels in serum, the worse the prognosis. Perinatal HPP is the most severe form, which can be lethal in utero or result in death soon after birth. Common clinical presentation is presence of nearly complete demineralization of skeleton. Other manifestations include lung hypoplasia, irritability, high-pitched cry, and pyridoxine-dependent seizures. The radiographic findings are confirmatory and include unmineralized skeleton and pathognomonic metaphyseal tongues of radiolucency. Infantile HPP manifests after birth but before 6 months of age when failure to thrive or delaying of milestones along with rickets appear. Skeletal deformities, rib fractures, and trachemalacia leads to death of approximately 50% of patients in infancy, mostly secondary to pneumonia. Presence of pyridoxine-dependent seizures is associated with worst prognosis. Childhood (juvenile) HPP presents after 6 months of age with a wide range of clinical symptoms. Premature loss of deciduous teeth are very common. Muscle weakness and delayed walking with a waddling gait are some other clinical presentations. The clinical manifestations of childhood HPP are present during growth but sometimes symptoms improve during young adult life and may reoccur later in life. Adult HPP usually manifests in middle age with loss of adult dentition, nonhealing metatarsal fractures, and femoral pseudofractures (proximal and lateral subtrochanteric region).^3,4^ Some adults may give a history of premature loss of deciduous teeth and rickets in childhood. Accumulation of inorganic pyrophosphate extracellularly can cause calcium pyrophosphate dehydrate deposition, pseudogout, and calcific periarthritis.^3,5^ Skeletal and joint pain is also common secondary to recurrent fractures.^5^ Patient with adult HPP are also prone to develop atypical femoral fractures, which involve proximal and lateral subtrochanteric regions, which are similar to fractures developed as adverse reaction to antiresorptive medications (bisphosphonates). These fractures usually cause chronic hip pain and predispose morbidity associated with this untreatable disease.^4^

Benign perinatal HPP is similar to perinatal HPP in clinical findings but skeletal manifestations seen in utero improved postnatally. Pseudohypophosphatasia is an extremely rare entity and resembles infantile HPP, though serum ALP activity is normal or increased.^3^ Odontohypophosphatasia is the least severe form, which manifests with dental complications without any clinical or radiographic evidence of rickets or osteomalacia.^2,3^ Premature loss of deciduous teeth is the most common presentation.

Diagnosis relies on clinical presentation, radiographic abnormalities, and low ALP level. Genetic testing for TNSALP mutation is not necessary in making the diagnosis but can be helpful in prenatal assessment.^2,3^ It is important to use an age- and sex-specific range of ALP, as using adult values can miss diagnosis of HPP in infants and children. Elevated serum pyridoxal 5′-phosphate (active form of vitamin B_6_) is the most sensitive and specific marker to diagnose HPP, and the degree of elevation usually correlates with the severity of the illness.^3,8^ Elevated levels of urinary or serum phosphoethanolamine and inorganic pyrophosphate also support the diagnosis of HPP.^9,10^

The treatment of HPP is usually supportive. Severely affected babies may require mechanical ventilation due to chest deformities. Vitamin B_6_–dependent seizures can respond to administration of pyridoxine. Good dental care is also important as loss of teeth can cause impairment of speech and nutrition.^3^ Unlike other forms of osteomalacia, calcium and vitamin D supplementation is usually not recommended as the levels of calcium and vitamin D is usually normal in these patients.^3^ Therefore, supplementing calcium and vitamin D can predispose to hypercalcemia hypercalciuria and vitamin D toxicity.^3^ In adults, treatment includes foot orthoses for metatarsal fractures, nonsteroidal anti-inflammatory drugs for CPPD (calcium pyrophosphate crystal deposition Disease), and intramedullary fixation for femoral fractures. Use of soluble ALP and bone cell transplantation has been studied in the past but was unsuccessful.^11-14^ Some adults benefit from the use of teriparatide in the past but the use is controversial. Bisphosphonates are generally not indicated in the treatment.^15,16^

Asfotase alfa (Strensiq), which is bone-targeted human recombinant TNSALP replacement therapy, has been recently approved in the United States for the treatment of perinatal/infantile or juvenile onset HPP. In a study of 11 infants and children, use of asfotase alfa has been associated with improved pulmonary function, developmental milestones, and decrease in levels of TNSALP substrates (pyridoxal phosphate and inorganic pyrophosphate).^17^ Subsequent studies also showed asfotase alfa to be associated with improved overall survival.^18^

HPP is associated with high mortality especially in pediatric patients, in which mortality rates range from almost 100% in perinatal HPP to over 50% in infantile onset. Adult HPP is debilitating due to recurrent metatarsal fractures, femoral fractures, and skeletal and bone pain.^3-5^ Recent Food and Drug Administration approval of asfotase alfa for infantile and juvenile HPP is believed to decrease mortality and morbidity associated with this last form of osteomalacia to await medical treatment.

## Conclusion

Clinicians should be attentive to a history of recurrent low-trauma fractures in adults, premature loss of deciduous teeth, and persistently low serum ALP to suspect this diagnosis. Early detection with the availability of enzyme-replacement therapy may avoid years of undiagnosed morbidity.
